# Systematic Exploration in Tissue-Pathway Associations of Complex Traits Using Comprehensive eQTLs Catalog

**DOI:** 10.3389/fdata.2021.719737

**Published:** 2021-11-03

**Authors:** Boqi Wang, James Yang, Steven Qiu, Yongsheng Bai, Zhaohui S. Qin

**Affiliations:** ^1^ Emory University, Atlanta, GA, United States; ^2^ Carmel High School, Carmel, IN, United States; ^3^ James Martin High School, Arlington, TX, United States; ^4^ Next-Gen Intelligent Science Training, Ann Arbor, MI, United States; ^5^ Department of Biostatistics and Bioinformatics, Emory University, Atlanta, GA, United States

**Keywords:** eQTLs, gene pathway sets, gene set enrichment analyses, tissue-pathway association, complex traits

## Abstract

The collection of expression quantitative trait loci (eQTLs) is an important resource to study complex traits through understanding where and how transcriptional regulations are controlled by genetic variations in the non-coding regions of the genome. Previous studies have focused on associating eQTLs with traits to identify the roles of trait-related eQTLs and their corresponding target genes involved in trait determination. Since most genes function as a part of pathways in a systematic manner, it is crucial to explore the pathways’ involvements in complex traits to test potentially novel hypotheses and to reveal underlying mechanisms of disease pathogenesis. In this study, we expanded and applied loci2path software to perform large-scale eQTLs enrichment [i.e., eQTLs’ target genes (eGenes) enrichment] analysis at pathway level to identify the tissue-specific enriched pathways within trait-related genomic intervals. By utilizing 13,791,909 eQTLs cataloged in the Genotype-Tissue Expression (GTEx) V8 data for 49 tissue types, 2,893 pathway sets reported from MSigDB, and query regions derived from the Phenotype-Genotype Integrator (PheGenI) catalog, we identified intriguing biological pathways that are likely to be involved in ten traits [Alzheimer’s disease (AD), body mass index, Parkinson’s disease (PD), schizophrenia, amyotrophic lateral sclerosis, non-small cell lung cancer (NSCLC), stroke, blood pressure, autism spectrum disorder, and myocardial infarction]. Furthermore, we extracted the most significant pathways for AD, such as BioCarta D4-GDI pathway and WikiPathways sulfation biotransformation reaction and viral acute myocarditis pathways, to study specific genes within pathways. Our data presented new hypotheses in AD pathogenesis supported by previous studies, like the increased level of caspase-3 in the amygdala that cleaves GDP dissociation inhibitor and binds to beta-amyloid, leading to increased apoptosis and neuronal loss. Our findings also revealed potential pathogenesis mechanisms for PD, schizophrenia, NSCLC, blood pressure, autism spectrum disorder, and myocardial infarction, which were consistent with past studies. Our results indicated that loci2path′s eQTLs enrichment test was valuable in unveiling novel biological mechanisms of complex traits. The discovered mechanisms of disease pathogenesis and traits require further in-depth analysis and experimental validation.

## 1 Introduction

Expression quantitative trait loci (eQTLs) have been one of the major focuses in determining the genetic variants that affect gene expressions locating in non-coding regions of the genome. eQTLs’ nature of influencing expression levels of their target genes (eGenes) makes them powerful at studying transcription regulation (Li et al., 2010). The traditional usage of genomic physical proximity to connect genetic loci with their corresponding eGenes has been proven somewhat ineffective since it has been demonstrated that only about 25% of eQTLs have their physically closest genes to be their eGenes (Zhu et al., 2016; Xu et al., 2020). Further, eQTLs have become an increasingly popular tool for researchers to identify specific genes for diseases and traits.

Researchers often use eQTLs associations to link expression traits to genotypes of genetic variants located in genomic intervals. Multiple studies have been conducted on connecting eQTLs and various traits including Alzheimer’s disease (AD) to determine the roles trait-related eQTLs and their corresponding eGenes play in pathogenesis (Hormozdiari et al., 2016; Zhao et al., 2019; Sieberts et al., 2020). Though many interesting findings have been discussed, the observed eQTLs patterns in cerebral and cerebellar brain regions require further investigations with respect to their potential functions, but so far, to our knowledge, no systematic in-depth studies have been performed to explore the roles of such eQTLs in etiologies of neurodegenerative diseases such as AD (Zhao et al., 2019; Sieberts et al., 2020). Another common practice is to use eQTLs mapping to link an expression trait to genetic variants in certain genomic regions, which holds promise in elucidating gene regulations and predicting gene networks associated with complex phenotypes (Li et al., 2010). By using eQTLs mapping methods, we can generate a comprehensive connection map of eQTLs and their eGenes’ enriched pathways to help us develop a more thorough understanding of eQTLs’ involvement in gene regulation, thus providing insights in discovering hidden biological mechanisms (Gilad et al., 2008). In addition, eQTLs studies can also help reveal the architecture of gene regulation, which in combination with results from previous genetic association studies of human traits may help predict regulatory roles for genetic variants previously associated with particular human phenotypes (Gilad et al., 2008). Therefore, it is crucial to explore the associations between eQTLs and genes at the pathway level in complex traits to develop a systematic review of such associations and infer mechanisms of pathogenesis.

The objective of this study was to perform large-scale eQTLs enrichment tests at the pathway level and determine the tissue-specific enriched pathways for trait-related genomic intervals based on the Bioconductor package loci2path (Xu et al., 2020). There are two key advantages of using loci2path than other existing methods: first, we do not depend on physical proximity to provide a link between an eQTL and its target gene, which could be unreliable; second, eQTLs enable us to produce the regulatory annotation for specific tissue types (Xu et al., 2020). For a specific genomic interval containing multiple eQTLs, if eQTLs enrichment analysis indicates that their corresponding eGenes are participating in the same biological pathway, this could imply a potential relationship between that specific pathway and the genomic interval of interest. The tissue-specific eQTLs sets also can demonstrate in what specific tissues would such enrichment be observed, which could help us generate new hypotheses on the biological mechanisms of disease pathogenesis.

In this study, we used the computer program loci2path to perform eQTLs enrichment analysis for genomic regions of ten traits [AD, body mass index, Parkinson’s disease (PD), schizophrenia, amyotrophic lateral sclerosis, non-small cell lung cancer (NSCLC), stroke, blood pressure, autism spectrum disorder, and myocardial infarction]. We have updated the loci2path to utilize the most current data sets of query regions, eQTLs sets, and pathway sets. We used the entire multi-tissue eQTLs data from the GTEx V8 data release that contains 13,791,909 eQTLs with 32,958 unique eGenes for 49 tissue types. In addition to BioCarta and Kyoto Encyclopedia of Genes and Genomes (KEGG) pathway sets that were included in the original loci2path (Xu et al., 2020), we have added pathway sets from three new pathway databases, i.e., Pathway Interaction Database (PID), Reactome, and WikiPathways to generate more comprehensive results.

## 2 Materials and Methods

### 2.1 Extension of the loci2path

In this study, we extended the Bioconductor package loci2path (Xu et al., 2020) that runs on an R-based platform, and then applied the extended loci2path to perform eQTLs enrichment analyses at pathway level based on different pathway databases to identify enriched pathways for genomic intervals of multiple traits. The advantage of loci2path is that this computer program uses eQTLs information to directly link to their eGenes, rather than using genome proximity, because an eQTL and its corresponding eGene are not always located near each other. For each gene set, the loci2path will first identify eGenes based on the eQTLs set in the given genomic intervals and then evaluate the significance of these eGenes’ enrichment within a gene set. The eQTLs enrichment program really refers to their corresponding eGenes’ enrichment because multiple eQTLs could target the same eGenes due to linkage disequilibrium. *p*-values calculated using Fisher’s exact test for an eQTLs set could be computed for each pathway to evaluate the enrichment significance, and those pathways with greater enrichments were indicated by smaller *p*-values. The results were filtered with a *p*-value of 10^−4^, which was chosen after multiple trials to balance the number of most significant tissue-pathway combinations and specificity, and used to construct heatmaps for further analysis. We have tried other *p*-values and obtained similar outcomes.

### 2.2 Datasets

#### 2.2.1 GTEx eQTLs

For this study, we used the full set of multi-tissue QTL data from the GTEx V8 data release as the input data of eQTLs sets, consisting of 49 tissue types (GTEx Consortium, 2020). The data were downloaded from GTEx through this link: https://storage.googleapis.com/gtex_analysis_v8/multi_tissue_qtl_data/GTEx_Analysis_v8.metasoft.txt.gz. eQTLs sets for each tissue were filtered with a *p*-value threshold of 10^−4^. Each gene’s entrez ID and gene name were obtained by using the given gene’s ensemble gene ID and the Bioconductor package biomaRt.

#### 2.2.2 MSigDB Pathways

A total of 2,893 pathways from BioCarta, KEGG, PID, Reactome, and WikiPathways gene sets were used in this study as the input data of gene sets. The data were downloaded from the MSigDB website: http://www.gsea-msigdb.org/gsea/msigdb/collections.jsp.

#### 2.2.3 Phenotype-Genotype Integrator Query Regions

The list of known trait-associated variants was obtained from National Center for Biotechnology Information (NCBI) via PheGenI website: https://www.ncbi.nlm.nih.gov/gap/phegeni. For a given genetic variant, the genomic region is defined as a flanking 50 kb on each of left and right sides of that variant, which spans 100 kb. Overlapped regions were merged. A total of 9,894 genomic intervals were used in this study, and the numbers of genomic regions for each trait are demonstrated in Table 1.

**TABLE 1:** The numbers of genomic intervals selected that contain known GWAS variants for each of the ten complex traits.

Trait	Number of genomic intervals
Alzheimer’s Disease	319
Body Mass Index	2,052
Parkinson’s Disease	199
Schizophrenia	1,296
Amyotrophic Lateral Sclerosis	342
Non-Small Cell Lung Cancer	120
Stroke	939
Blood Pressure	3,123
Autism Spectrum Disorder	570
Myocardial Infarction	934

## 3 Results

### 3.1 Overview

**FIGURE 1:**
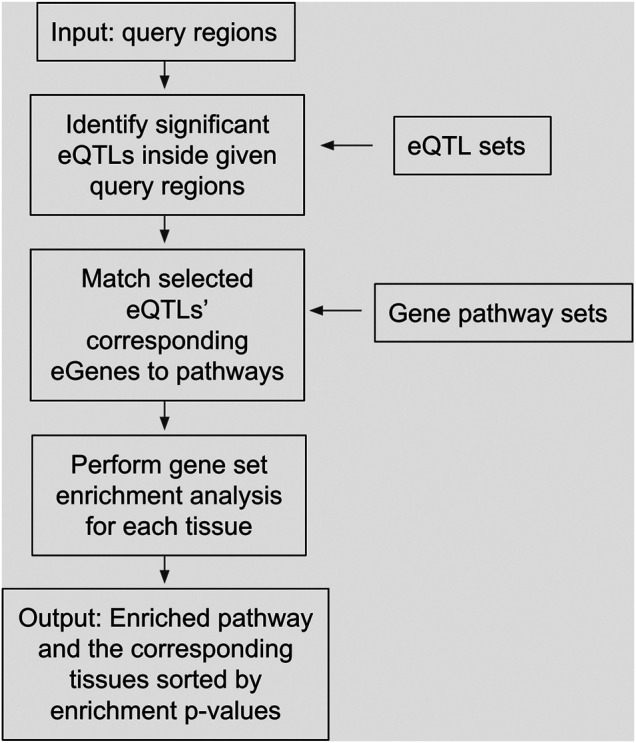
A diagram depicting our study’s analysis pipeline, including input data, internal processes, and output results.

The objectives of this study were to identify significantly enriched pathways for eQTLs sets of specific tissues at trait-related genomic intervals to generate potentially novel hypotheses of trait determination. A workflow of the study is presented in Figure 1, showing that the input data were query regions, and the internal process involved usages of eQTLs sets and gene pathway sets, and the output results were enriched pathways and the corresponding tissues sorted by multiplicity-adjusted enrichment *p*-values. We used loci2path to conduct eQTLs enrichment analyses by computing the *p*-values of Fisher’s exact test adjusted by Benjamini & Hochberg correction method (Benjamini & Hochberg, 1995), and then converting such results into a heatmap. The heatmap was displayed where each row represents a tissue type, and each column represents a gene pathway. The strong significant enrichments were indicated by red cells, and the weak insignificant enrichments were indicated by blue cells. Other data including eQTLs in pathways, eQTLs in tissues, and hit genes generated by loci2path were used to construct tables. Various adjusted *p*-values of genes through Fisher’s exact test were used as thresholds to filter out the most significant pathway-tissue combinations for each trait. Specific genes that pathways hit in the eQTLs sets were extracted for further analysis. Additional heatmaps and result tables for traits can be found in Supplementary Figures. The results of three of the ten traits, i.e., body mass index, amyotrophic lateral sclerosis, and stroke were not presented, because the outputs obtained from eQTLs enrichment tests at the pathway level for these traits were insignificant, and no further analyses could be performed on them.

### 3.2 Adding PID, Reactome, and WikiPathways to loci2path

We have extended the loci2path (Xu et al., 2020) by adding gene pathway sets of PID, Reactome, and WikiPathways to loci2path′s pathway collection. The data of pathway links and NCBI entrez gene IDs were retrieved from the MSigDB website: http://www.gsea-msigdb.org/gsea/msigdb/collections.jsp. The data were separated into two text documents with one containing gene links and the other containing the pathway’s gene entrez IDs using a self-written R program (Supplementary Data Sheet S1). The documents were added into the loci2path Bioconductor package at loci2path-master/inst/extdata/geneSet, which could be called by the loci2path-running program to match significant eQTLs at the new gene pathway sets.

### 3.3 Alzheimer’s Disease

Currently, there are three major pathology divisions for AD: protein accumulation, neuron loss, and reactive process (Duyckaerts et al., 2009). Past studies have shown that the extracellular accumulation and deposition of amyloid-beta (Aβ) protein induce the appearance of senile plaques and create an abnormal neuron environment, which causes cognitive disabilities (Sadigh-Eteghad et al., 2015; Cheignon et al., 2018). Such accumulation of Aβ not only enhances the interaction between amyloid-forming protein and neuronal membrane and increases membrane permeability through hypothetical mechanisms like amyloid-forming protein’s channel-like conductance, but also contributes to the increase in the reactive oxygen species production and thus the disruption of neuronal membrane integrity (Butterfield and Lashuel, 2010; Cheignon et al., 2018).

**FIGURE 2:**
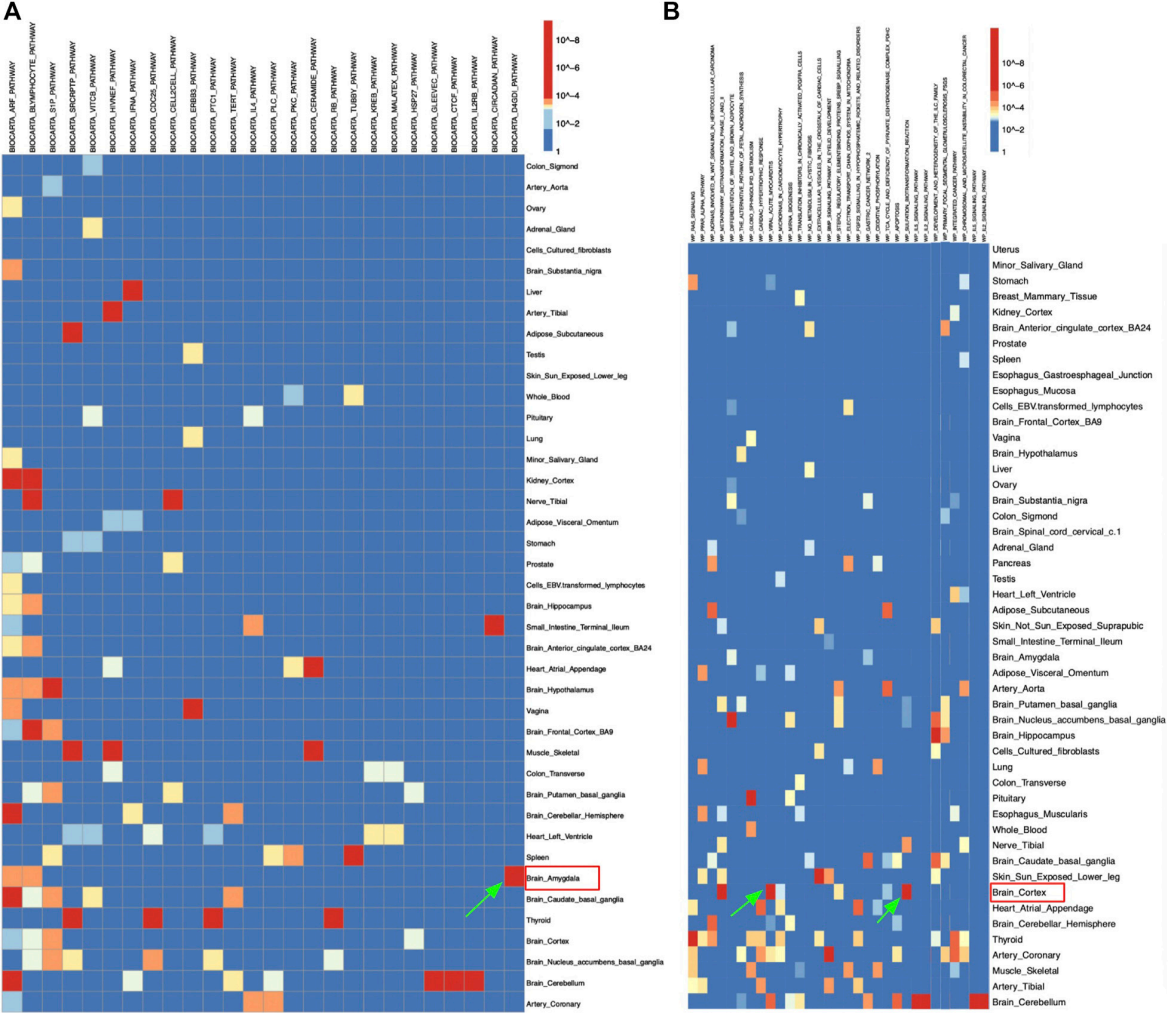
Heatmap of Alzheimer’s disease’s eQTLs enrichment results in **(A)** BioCarta and **(B)** WikiPathways pathway sets, respectively.

Figure 2A demonstrated the eQTLs enrichment of AD-related genomic intervals in the BioCarta pathway set. There was a distinct significant enrichment of the D4-GDI pathway in the brain amygdala (Figure 2A). Significant eQTLs enrichment results from the amygdala tissue were extracted for further analysis. The table has demonstrated that most pathways’ gene hit in brain amygdala tissue was Rho GDP dissociation inhibitor beta (*ARHGDIB*) gene (Table 2). The D4-GDI pathway had the lowest *p*-value of genes, which was consistent with the data in Figure 2A where the D4-GDI pathway was only enriched in amygdala tissue (Table 2; Figure 2A). D4-GDI represents the negative regulator of Ras-related Rho GTPases, and its removal is crucial to induce apoptosis since Rho GTPases increase the cytoskeletal and membrane modification related to apoptosis (Coleman and Olson, 2002). As an enzyme that cleaves D4-GDI, caspase-3 was found to be positively correlated with mild cognitive deficiency in early AD pathology (Gastard et al., 2003). Clinical research suggested that Aβ could sequester caspase-3 via direct interaction and induce neuronal apoptosis via caspase-3 activation, thus strengthening AD development (Chang et al., 2016). One possible hypothesis was that an increased level of caspase-3 in the amygdala leads to increased apoptosis and neuronal loss and thus contributes to the memory loss symptom of AD.

**TABLE 2:** P-values Obtained from Fisher’s Exact Test of Significant eQTLs Enrichment for Alzheimer’s Disease in BioCarta Pathway Set for Brain Amygdala Tissue

Pathway	Gene hit	Genomic location	Fisher’s exact test *p*-value^a^
D4-GDI	*ARHGDIB*	12p12.3	0.020
Blymphocyte	*CR1*	1q32.3	0.023
ARF	*POLR1A*	2p11.2	0.028
Caspase	*ARHGDIB*	12p12.3	0.037
TNFR1	*ARHGDIB*	12p12.3	0.048
FAS	*ARHGDIB*	12p12.3	0.050
HIVNEF	*ARHGDIB*	12p12.3	0.091

a Fisher’s exact test *p*-value represents the adjusted *p*-value for genes in the pathway using Fisher’s exact test that are adjusted by Benjamini & Hochberg correction method.

Similarly, Figure 2B showed significant enrichment of sulfation biotransformation reaction and viral acute myocarditis pathways in brain cortex, IL2 and IL5 signaling pathways in brain cerebellum, and development and heterogeneity of the innate lymphoid cell (ILC) pathway in brain hippocampus for the WikiPathways set (Figure 2B). The significant enrichment of viral acute myocarditis pathway in the brain cortex suggested that the correlation observed between heart failure and AD was due to not only the majority of patients’ age, but also genetic factors (Figure 2B) (Li et al., 2006). Such findings were consistent with a previous study where the viral myocarditis pathway from other pathway sets was identified to be significantly associated with AD (Liu et al., 2014). One population study also found a higher than 80% risk of developing AD for patients with heart failures when major confounders like vascular comorbidities were controlled (Qiu et al., 2006). The significant enrichment in the sulfation biotransformation reaction pathway could also be explained by previous findings (Figure 2B). One research suggested an increased frequency of reduced metabolism and impaired sulfation of xenobiotics among AD patients (McFadden, 1996). A clinical study showed that sulfated curcumin can bind to copper and iron ions that are enriched in the brain cortex of AD patients and induce Aβ peptide formation, thus indicating that impaired sulfation ability would increase risk of AD (Baum and Ng, 2004). One possible connection between acute viral myocarditis and AD is kynurenine 3-monooxygenase (KMO), which is a key regulatory enzyme in the kynurenine metabolism pathway that converts kynurenine to 3-hydroxykynurenine (Kubo et al., 2017). Studies have shown that the absence of KMO increased the production of kynurenine pathway metabolite, which lowered the synthesis of chemokine and thus resulted in the decrease of mortality of viral acute myocarditis by encephalomyocarditis virus in mice (Kubo et al., 2017). Interestingly, another study pointed out that JM6, a KMO inhibitor, was found to be able to prevent memory deficiency and synaptic loss in AD mouse models through the increase of the neuroprotective kynurenine metabolite kynurenic acid (Zwilling et al., 2011). Such interaction may imply a hidden mechanism in AD’s pathogenesis that increases KMO production and thus decreases levels of neuroprotective kynurenine metabolite and enhances AD symptoms, which explains AD’s connection to acute viral myocarditis.

### 3.4 Parkinson’s Disease

One key sign of PD is the accumulation of α-synuclein and the formation of Lewy bodies in brainstem, limbic system, and cortical areas (Alecu and Bennett, 2019). Pathological hallmarks also include the loss of dopaminergic neurons from the substantia nigra and Lewy bodies in surviving cells of affected brains, which leads to reduced voluntary movements (Gegg et al., 2012).

As demonstrated in the Supplementary Figure S1A, the enrichment of the KEGG sphingolipid metabolism pathway was observed to be highly and uniquely significant in amygdala tissue, which indicates a correlation between sphingolipid metabolism and PD. This is consistent with previous studies since the metabolism of sphingolipid glucosylceramide catalyzed by glucocerebrosidase (GCase) was found to be deficient in PD patients (Gegg et al., 2012). The deficiency of GCase that catalyzes sphingolipid metabolism has reached up to 40% at amygdala for PD patients compared to normal patients, which is likely to cause α-synuclein accumulation as GCase mRNA level decreased in cells with exogenous α-synuclein (Gegg et al., 2012). One possible explanation for the decreasing GCase could be a mutation at glucosylceramidase-beta gene that encodes this lysosomal enzyme. Similarly, the lysosomal-associated membrane protein 2A and heat shock cognate 70 from lysosome had significantly lower expression levels in amygdala of brains with PD compared to brains with AD or normal brains (Alvarez-Erviti et al., 2010). The chaperone-mediated autophagy strongly depends on these two proteins, and the downregulation of lysosomal-associated membrane protein 2A has increased the mean half-life of α-synuclein from 46.5 to 65 h, suggesting a direct link between this protein and PD (Alvarez-Erviti et al., 2010). Since wild-type α-synuclein was mostly degraded by chaperone-mediated autophagy, it is valid to hypothesize that impaired lysosomal functions could initiate the accumulation of α-synuclein and thus lead to PD.

### 3.5 Schizophrenia

**FIGURE 3:**
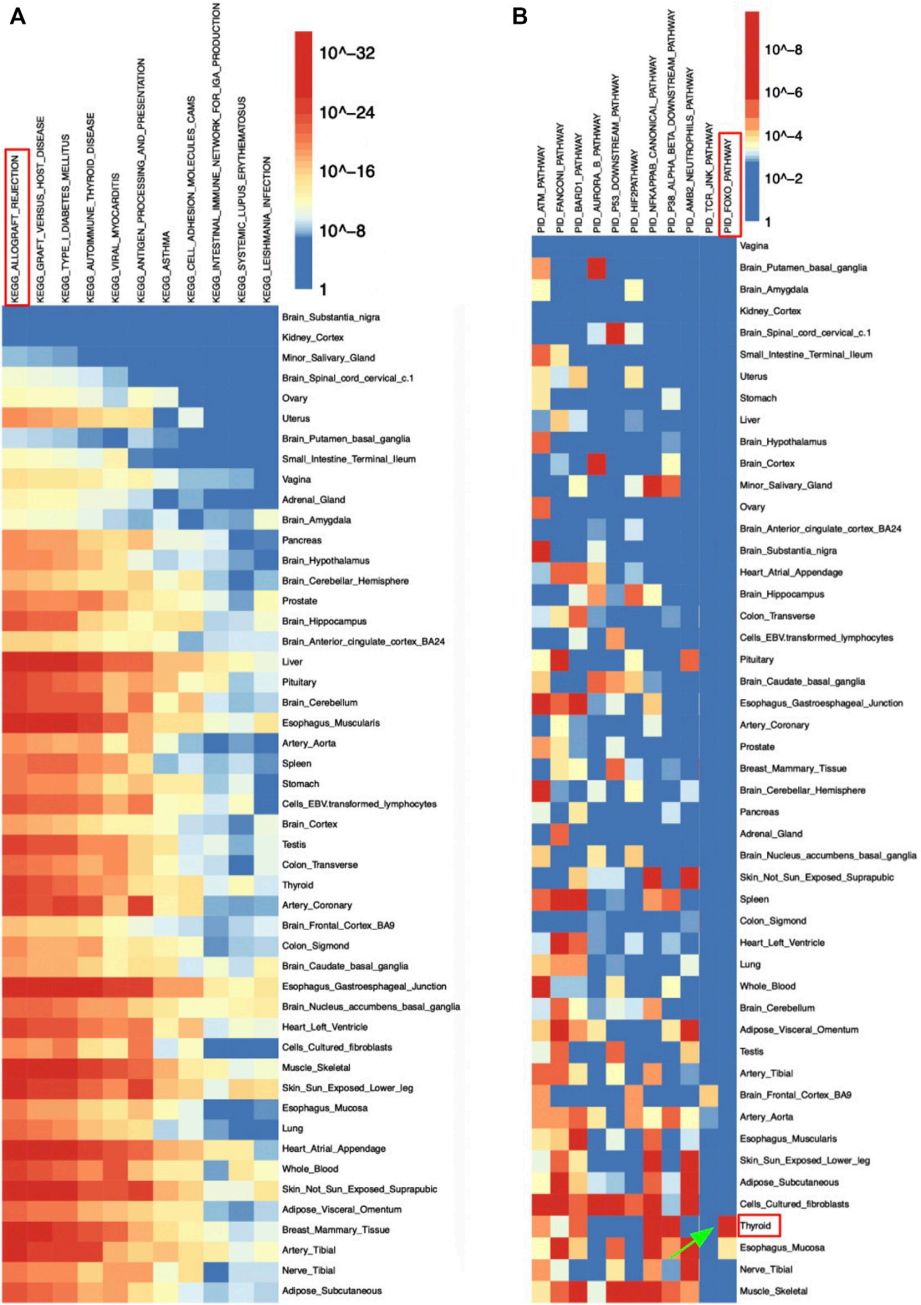
Heatmap of schizophrenia’s eQTLs enrichment results in **(A)** KEGG and **(B)** PID pathway sets.

As demonstrated, most significantly enriched pathways in all 49 tissues were immune-related pathways including allograft rejection, graft vs. host disease, and antigen processing and presentation pathways (Table 3). The significantly enriched KEGG allograft rejection pathway in different tissues shared the major histocompatibility complex, Class I, C (*HLA-C*) gene (Figure 3A; Table 3). *HLA-C* has been shown to be strongly associated with schizophrenia by multiple past studies. HLA-C*01:02 was positively associated with schizophrenia, while HLA-C*07:01 was negatively associated with schizophrenia (Andreassen et al., 2015; Corvin, 2012). One study suggested that in the absence of glutamic acid at the 74th position of the mature protein encoded by the major histocompatibility complex, Class II, DR Beta 1(*HLA-DRB1*) gene, the amino acid methionine at the 99th position of *HLA-C* may contribute to individuals’ susceptibility to schizophrenia, in which the glutamic acid in *HLA-DRB1* has a protective function against the disease (Seshasubramanian et al., 2020). Interestingly, *HLA-DRB1* was hit by the majority of tissues enriched with the KEGG allograft rejection pathway (Table 3). Similarly, the major histocompatibility complex, Class II, DQ Beta 1 (*HLA-DQB1*) gene was also shared by most tissues with such a pathway, a molecule that presents peptides derived from extracellular proteins and is expressed in antigen expression cells (Table 3). DQB1*05:01:01 was also positively associated with schizophrenia and the predominant haplotype in the schizophrenia population, while decreased frequency of DQB1*02:01 was found among schizophrenia patients (Katrinli et al., 2019; Seshasubramanian et al., 2020). No studies have been conducted on specific mechanisms of *HLA-C*, *HLA-DRB1*, and *HLA-DQB1*’s interventions in schizophrenia pathogenesis, but their interaction is much likely to contribute to the disease.

**TABLE 3:** Adjusted *p*-values of the Ten Most Significant eQTLs for Schizophrenia from 49 tissues.

Tissue	Pathway	Gene hits	Genomic locations	Fisher’s exact test *p*-value^a^
Breast Mammary Tissue	KEGG allograft rejection	*CD80;HLA-E;HLA-G;HLA-C;HLA-DQB1;HLA-DRB5;HLA-DOB;HLA-DQA2;HLA-DRB1;HLA-DQA1;HLA-DRA;HLA-B*	3q13.33, 6p22.1, 6p21.33, 6p21.32	2.59E-12
	KEGG graft versus host disease	*CD80;HLA-E;HLA-G;HLA-C;HLA-DQB1;HLA-DRB5;HLA-DOB;HLA-DQA2;HLA-DRB1;HLA-DQA1;HLA-DRA;HLA-B*	3q13.33, 6p22.1, 6p21.33, 6p21.32	1.05E-11
	KEGG type I diabetes mellitus	*CD80;HLA-E;HLA-G;HLA-C;HLA-DQB1;HLA-DRB5;HLA-DOB;HLA-DQA2;HLA-DRB1;HLA-DQA1;HLA-DRA;HLA-B*	3q13.33, 6p22.1, 6p21.33, 6p21.32	1.99E-11
Esophagus Gastroesphageal Junction	KEGG type I diabetes mellitus	*CD80;HLA-E;HLA-G;HLA-A;HLA-C;HLA-DQB1;HLA-DRB5;HLA-DQA2;HLA-DMA;HLA-DRA;HLA-DRB1;HLA-DQA1;HLA-B;LTA*	3q13.33, 6p22.1, 6p21.33, 6p21.32	1.59E-14
	KEGG allograft rejection	*CD80;HLA-E;HLA-G;HLA-A;HLA-C;HLA-DQB1;HLA-DRB5;HLA-DQA2;HLA-DMA;HLA-DRA;HLA-DRB1;HLA-DQA1;HLA-B*	3q13.33, 6p22.1, 6p21.33, 6p21.32	6.37E-14
	KEGG graft versus host disease	*CD80;HLA-E;HLA-G;HLA-A;HLA-C;HLA-DQB1;HLA-DRB5;HLA-DQA2;HLA-DMA;HLA-DRA;HLA-DRB1;HLA-DQA1;HLA-B*	3q13.33, 6p22.1, 6p21.33, 6p21.32	3.00E-13
	KEGG antigen processing and presentation	*CTSS;HLA-E;HLA-G;HLA-A;HLA-C;HLA-DQB1;HLA-DRB5;HLA-DQA2;HLA-DMA;HLA-DRA;HLA-DRB1;HLA-DQA1;TAP2;TAPBP;HLA-B;LTA*	6p22.1, 6p21.33, 6p21.32, 1q21.3	2.52E-12
	KEGG autoimmune thyroid disease	*CD80;HLA-E;HLA-G;HLA-A;HLA-C;HLA-DQB1;HLA-DRB5;HLA-DQA2;HLA-DMA;HLA-DRA;HLA-DRB1;HLA-DQA1;HLA-B*	3q13.33, 6p22.1, 6p21.33, 6p21.32	9.45E-12
Muscle Skeletal	KEGG allograft rejection	*CD80;HLA-E;HLA-C;HLA-G;HLA-DQB1;HLA-DRB5;HLA-DMA;HLA-DRA; HLA-DQA2;HLA-DRB1;HLA-DQA1;HLA-A, HLA-B*	3q13.33, 6p22.1, 6p21.33, 6p21.32	5.14E-12
	KEGG graft versus host disease	*CD80;HLA-E;HLA-C;HLA-G;HLA-DQB1;HLA-DRB5;HLA-DMA;HLA-DRA; HLA-DQA2;HLA-DRB1;HLA-DQA1;HLA-A, HLA-B*	3q13.33, 6p22.1, 6p21.33, 6p21.32	2.37E-11

a Fisher’s exact test *p*-value represents the adjusted *p*-value for genes in the pathway using Fisher’s exact test that are adjusted by Benjamini & Hochberg correction method.

In the PID pathway set, the FOXO pathway was significantly enriched in the eQTLs set of thyroid tissue, which suggested a potential correlation between the forehead box transcription factor O family and schizophrenia at thyroid (Figure 3B). The mRNA expression level of FOXO pathway genes including *FOXO1* and *FOXO3A* were significantly lower in patients with acute schizophrenia (Gu et al., 2021).

### 3.6 Non-Small Cell Lung Cancer

The BioCarta IL1R pathway was shown to be significantly enriched in the testis tissue for NSCLC (Supplementary Figure S2). The IL1R pathway involves signal transduction through interleukin-1. One study found that interleukin-17 (IL-17) was involved in angiogenesis in a variety of inflammatory associated cancers, although it still remains unclear how IL-17 contributes to the process (Pan et al., 2015). It is also known that interleukin-37 (IL-37), a new member of the interleukin-1 family, plays an immunosuppressive role in a variety of inflammatory disorders. A study recently found that IL-37 demonstrates a protective role in cancer development possibly through tumor angiogenesis and that it could serve as a promising therapeutic target for NSCLC (Ge et al., 2016).

In Supplementary Figure S2, the PCG1A pathway was enriched in the kidney. The PCG1A pathway involves the regulation of peroxisome proliferator-activated receptor gamma coactivator-1 alpha (PGC-1a), which is a tissue-specific coactivator that enhances the activity of many nuclear receptors and coordinates transcriptional programs important for energy metabolism and homeostasis. In NSCLC patients, there are a variety of cases where the cells show therapeutic resistance. As a result, a plethora of studies focus on drug resistance mechanisms, but not many have focused on the metabolic flexibility of drug-resistant NSCLC. In one study, it was found that during the development of resistance for tyrosine kinase inhibitors, NSCLC cells switched from glycolysis to oxidative phosphorylation through increasing activity of the mitochondria. Cells were treated with the MCT-1 inhibitor AZD3965 and there was a resulting significant decrease in cell proliferation and motility in TK1-sensitive and TK-resistant cells. A study recently found that IL-37 demonstrates a protective role in cancer development possibly through tumor angiogenesis and that it could serve as a promising therapeutic target for NSCLC (Huang et al., 2020).

### 3.7 Blood Pressure

For blood pressure, the majority of the pathways most significantly enriched in tissues were immune-related, and the atrial appendages tissue contained the most pathways with the most significant *p*-values (Table 4). The role of the immune system in the pathogenesis of hypertension has been firmly established by many laboratories. The KEGG viral myocarditis pathway and the tissue heart atrial appendage had one of the most significant *p*-values at 3.08E-14, the KEGG type I diabetes mellitus pathway was also significantly enriched at the atrial appendage tissue (Table 4).

**TABLE 4:** Adjusted *p*-values of Five Most Significant eQTLs for Blood Pressure in KEGG and WikiPathways Sets for Heart Atrial Appendage Tissue.

Pathway	Gene hits	Genomic locations	Fisher’s exact test *p*-value^a^
WikiPathways Ebola virus pathway on host	*MERTK;KPNA1;RFC1;ITGA2;HLA-G;HLA-A;HLA-C;HLA-B;HLA-E;HLA-DOA;HLA-DRB5;HLA-DQB2;HLA-DMA;HLA-DPA1;HLA-DRB1;HLA-DPB1;HLA-DQA2;HLA-F;HLA-DQB1;HLA-DOB;HLA-DQA1;HLA-DRA;RAC1;SCIN;CAV2;CAV1;CTSB;ITGB1;TPCN2;MFGE8;IQGAP1;NPC1;VPS16*	6p22.1, 6p21.33, 6p21.32, 2q13, 3q21.1, 4p14, 5q11.2, 7p22.1, 7p21.3, 7q31.2, 8p23.1, 10p11.22, 11q13.3, 15q26.1, 18q11.2, 20p13	3.64E-13
WikiPathways allograft rejection	*CASP9;CD55;CD86;CSCL8;PDGFRA;BHMT2;HLA-G;HLA-A; HLA-C;HLA-B;C4A;HLA-E;MICA;HLA-DOA;HLA-DRB5;HLA-DMA;HLA-DPA1;HLA-DRB1;HLA-DPB1; HLA-DQA2;HLA-F;HLA-DQB1;HLA-DOB;C4B;HLA-DQA1;HLA-DRA;LRRK2*	6p22.1, 6p21.33, 6p21.32, 1p36.21, 1q32.2, 3q13.33, 4q12, 5q14.1, 12q12	1.03E-12
KEGG allograft rejection	*CD86;HLA-G;HLA-A;HLA-C;HLA-B;HLA-E;HLA-DOA; HLA-DRB5;HLA-DMA;HLA-DPA1;HLA-DRB1;HLA-DPB1;HLA-DQA2;HLA-F;HLA-DQB1;HLA-DOB;HLA-DQA1;HLA-DRA*	6p22.1, 6p21.33, 6p21.32, 3q13.33	1.03E-12
KEGG viral myocarditis	*CASP9;CD55;CD86;HLA-G;HLA-A;HLA-C;HLA-B;HLA-E;HLA-DOA;HLA-DRB5;HLA-DMA;HLA-DPA1;HLA-DRB1;HLA-DPB1;HLA-DQA2;HLA-F;HLA-DQB1;HLA-DOB;HLA-DQA1;HLA-DRA;RAC1;CAV1;RAC3*	6p22.1, 6p21.33, 6p21.32, 7p22.1, 7q31.2, 1p36.21, 1q32.2, 3q13.33, 17q25.3	5.70E-12
KEGG graft versus host disease	*CD86;HLA-G;HLA-A;HLA-C;HLA-B;HLA-E;HLA-DOA; HLA-DRB5;HLA-DMA;HLA-DPA1;HLA-DRB1;HLA-DPB1; HLA-DQA2;HLA-F;HLA-DQB1;HLA-DOB;HLA-DQA1;HLA-DRA*	6p22.1, 6p21.33, 6p21.32, 3q13.33	9.82E-12

a Fisher’s exact test *p*-value represents the adjusted *p*-value for genes in the pathway using Fisher’s exact test that are adjusted by Benjamini & Hochberg correction method.

Myocarditis is a cardiac disease associated with inflammation and injury of the myocardium. It results from various etiologies, but coxsackievirus is considered the dominant etiological agent. Infiltrating macrophages have been proven as a pivotal pathological inflammatory cell subset in coxsackievirus induced viral myocarditis, however, the mechanisms involving initiation and promotion are still unknown (Zhang et al., 2017).

Type 1 diabetes is the autoimmune destruction of the insulin producing beta-cells. High blood pressure is a common symptom of diabetes because the high levels of glucose in the blood damage the blood vessels and lead to hypertension. One study found that the left atrium mechanical functions were impaired in patients with type 1 diabetes (Acar et al., 2009).

### 3.8 Autism Spectrum Disorder

Few significant pathways were uniquely enriched in one or two tissues for autism spectrum disorder as shown in Supplementary Figures S3–S7. KEGG pathways of drug metabolism by cytochrome p450 and metabolism of xenobiotics by cytochrome p450 were found to be enriched in various tissues and most significantly in the liver tissue (Supplementary Figure S4; Table 5). Out of 29 most significant pathway-tissue combinations passing the *p*-value threshold of 10^−4^, genes *GSTM3* and *GSTM5* were hit 24 times, followed by genes *GSTM1*, *GSTP1*, *GSTM4*, and *GSTM2* (Supplementary Table S1). The two most significantly enriched pathways, Reactome phase II conjugation of compounds and KEGG metabolism of xenobiotics by cytochrome p45 pathways, were in liver tissues, and they have both hit genes *GSTM2*, *GSTM3*, *GSTM4*, and *GSTM5*, which encode for multiple proteins from the glutathione S-transferase mu class (Table 5). The two pathways cover proteins functioning in pharmacological inactivation of chemicals and detoxification, and the mu class enzymes are known for their functions in detoxification of electrophilic compounds by conjugation with glutathione (Cheng et al., 2020). Therefore, such highly significant adjusted *p*-values suggested a key role glutathione S-transferase mu enzymes play in autism spectrum disorder (Table 5). Studies have shown that when exposed to chronic heavy metal and chemical xenobiotic pollution, patients with autism spectrum disorder demonstrated significantly higher total glutathione and oxidized glutathione in red blood cells (Faber et al., 2019). The study also believed that the elevated glutathione was a compensatory mechanism to the exposure of a high xenobiotic environment (Faber et al., 2019). However, such a mechanism could not deal with oxidative stress as the reduced to oxidized glutathione ratio was lower in autistic patients, which indicates a crucial role glutathione plays in the xenobiotic detoxification among patients with autism spectrum disorder (Faber et al., 2019; Bjørklund et al., 2020).

**TABLE 5:** Adjusted *p*-values of 10 Most Significant eQTLs for autism spectrum disorder from 49 tissues.

Tissue	Pathway	Gene hits	Genomic locations	Fisher’s exact test *p*-value^a^
Adipose Visceral Omentum	Reactome biological oxidations	*GSTM5;GSTM3;GSTM1;GSTM4;EPHX1;NCOA1;ABHD14B;UGT2A1;SULT1E1;SLC26A1;UGT2B7;UGT3A2;AIP;GSTP1;CES1;CYB5B;ALDH3A1*	1p13.3, 1q42.12, 2p23.3, 3p21.2, 4q13.3, 4p16.3, 3q13.2, 5p13.2, 11q13.2, 16q12.2, 16q22.1, 17p11.2	3.44E-06
Brain Anterior cingulate cortex BA24	WikiPathways photodynamic therapyinduced NFE2L2 NRF2 survival signaling	*GCLM;EPHX1;ABCC2;GSTP1;CES1;NQO1;SRXN1*	1q42.12, 11q13.2, 16q12.2, 1p22.1, 10q24.2, 16q22.1, 20p13	7.66E-06
Brain Caudate basal ganglia	KEGG steroid hormone biosynthesis	*SRD5A3;UGT2A1;UGT2B4;UGT2B15;SULT1E1;UGT2B28*	4q13.3, 4q12, 4q13.2	1.62E-05
Colon Transverse	KEGG metabolism of xenobiotics by cytochrome p450	*GSTM5;GSTM3;GSTM2;GSTM1;GSTM4;EPHX1;UGT2B4;GSTP1;ALDH3A1*	1p13.3, 1q42.12, 11q13.2, 17p11.2, 4q13.3	1.28E-05
Kidney Cortex	Reactome biological oxidations	*GSTM5;GSTM3;GSTM4;GSTM2;NCOA1;UGT2A1;UGT2B4;UGT2B15;SULT1E1;UGT2B28;UGT3A2*	1p13.3, 2p23.3, 4q13.3, 5p13.2, 4q13.2	1.23E-06
Liver	Reactome phase II conjugation of compounds	*GSTM5;GSTM3;GSTM4;GSTM2;UGT2A1;UGT2B4;UGT2B15;SULT1E1;UGT2B28;UGT3A2*	1p13.3, 4q13.3, 5p13.2, 4q13.2	1.73E-08
	KEGG metabolism of xenobiotics by cytochrome p450	*GSTM5;GSTM3;GSTM4;GSTM2;UGT2A1;UGT2B4;UGT2B15;UGT2B28*	1p13.3, 4q13.3, 4q13.2	7.30E-08
	KEGG metabolism of xenobiotics by cytochrome p450	*GSTM5;GSTM3;GSTM1;GSTM4;EPHX1;UGT2A1;UGT2B7;ALDH3B2;GSTP1;ALDH3A1*	1p13.3, 1q42.12, 4q13.3, 3q13.2, 11q13.2, 17p11.2	3.71E-06
Lung	KEGG pentose and glucuronate interconversion	*UGDH;UGT2B4;UGT2A1;DHDH*	4q13.3, 4p14, 19q13.33	7.16E-06
Skin Not Sun Exposed Suprapubic	KEGG drug metabolism cytochrome p450	*GSTM5;GSTM3;GSTM4;GSTM2;UGT2A1;UGT2B4;UGT2B15;UGT2B28*	1p13.3, 4q13.3, 4q13.2	9.15E-08

a Fisher’s exact test *p*-value represents the adjusted *p*-value for genes in the pathway using Fisher’s exact test that are adjusted by Benjamini & Hochberg correction method.

### 3.9 Myocardial Infarction

Supplementary Figure S8 demonstrated the eQTLs enrichment in BioCarta and Reactome pathway sets of myocardial infarction-related genomic intervals. The AT1R pathway from the BioCarta pathway set was significantly enriched in brain cortex tissue (Supplementary Figure S8A), and the cell cycle pathway from the Reactome pathway set was enriched in whole blood tissue (Supplementary Figure S8B), respectively. *RAC1* gene was hit by the BioCarta AT1R pathway at the brain cortex tissue, and *PPP2R5A* gene was hit by the Reactome cell cycle pathway at the whole blood tissue (Table 6). In myocardial infarction, the RAC1 protein in the brain cortex tissue paired with the BioCarta AT1R pathway was enriched. The RAC1 protein belongs to the RAS superfamily of small GTP-binding proteins. Members of this superfamily appear to regulate a diverse array of cellular events, including the control of cell growth, cytoskeletal reorganization, and the activation of protein kinases. In terms of myocardial infarction, the RAC1 protein serves as a small GTP-binding protein that regulates NADPH oxidase. NADPH oxidase is a reactive oxygen species (ROS) that contributes to heart failure, such as myocardial infarction. Failing of the myocardium in patients with dilated cardiomyopathy (DCM) and ischemic cardiomyopathy (ICM) is characterized by an upregulation of NADPH oxidase–mediated ROS release associated with increased RAC1 activity (Maack et al., 2003).

**TABLE 6:** Adjusted *p*-values of Five Most Significant eQTLs for Myocardial Infarction in BioCarta and Reactome Pathway Sets from 49 tissues.

Tissue	Pathway	Gene hits	Genomic locations	Fisher’s exact test *p*-value^a^
Brain Cortex	BioCarta AT1R pathway	*SHC1;AGT;RAC1;GNAQ;MAPK3*	1q21.3, 1q42.2, 7p22.1, 9q21.2, 16p11.2	0.00378
	BioCarta PYK2 pathway	*SHC1;MAPK14;RAC1;GNAQ;MAPK3*	1q21.3, 7p22.1, 9q21.2, 16p11.2, 6p21.31	0.00378
Brain Nucleus accumbens basal ganglia	Reactome glutathione conjugation	*GSTM2;GSTM5;GSTM1;HPGDS;GGCT;GSTO1;CNDP2*	1p13.3, 4q22.3, 7p14.3, 10q25.1, 18q22.3	3.71E-05
Lung	BioCarta ATRBRCA pathway	*RAD17;FANCE;FANCG;MRE11;FANCA*	5q13.2, 6p21.31, 9p13.3, 11q21, 16q24.3	0.00950
Ovary	BioCarta ATRBRCA pathway	*RAD17;FANCG;MRE11;FANCA*	5q13.2, 9p13.3, 11q21, 16q24.3	0.00385
Testis	Reactome signaling by Rho GTPases	*KDM1A;WASF2;YWHAQ;CENPC;RASGRF2;IQGAP2;H2BC1;H3C6;H2BC3;H2AC4;H2BC4;CENPQ;MAPK14;H3C12;RAC1;H2AZ2;ARHGEF35;ARHGEF10;DLC1;RHOBTB1;CFL1;KLC2;CTTN;RHOG;RHOJ;MAPK3;SKA1;SPC24;SRC*	1p36.12, 6q21, 2p25.1, 4q13.2, 7p22.1, 16p11.2, 6p21.31, 5q14.1, 5q13.3, 6p22.2, 6p12.3, 6p22.1, 7p13, 7q35, 8p23.3, 8p22, 10q21.2, 11q13.1, 11q13.2, 11q13.3, 11p15.4, 14q23.2, 18q21.1, 19p13.2, 20q11.23	8.38E-05
Whole Blood	Reactome cell cycle	*PPP2R5A;AHCTF1;LPIN1;VRK2;MZT2A;ANAPC4;CENPC;DHFR;H3C6;H4C3;H2BC5;CENPQ;TUBB2B;TUBB2A;CDKN1A;H4C12;H2BC14;POM121;MAD1L1;H2AZ2;POM121C;PRKAR2B;MCM4;RAB2A;DCTN3;CDKN2B;CDKN2A;SMC2;PPP2R2D;BANF1;RAB1B;MRE11;NUP98;ANKLE2;PSMC6;PPP2R5E;MAPK3;SPC24;CHMP4B;DSN1*	16p11.2, 11q21, 4q13.2, 6p22.2, 6p12.3, 7p13, 19p13.2, 1q32.3, 1q44, 2p25.1, 2p16.1, 2q21.1, 4p15.2, 5q14.1, 6p25.2, 6p21.2, 6p22.1, 7q11.23, 7p22.3, 7q22.3, 8q11.21, 8q12.1, 9p13.3, 9p21.3, 9q31.1, 10q26.3, 11q13.1, 11q13.2, 11p15.4, 12q24.33, 14q22.1, 14q23.2, 20q11.22, 20q11.23	1.61E-07
	Reactome Rho GTPase effectors	*WASF2;PPP2R5A;AHCTF1;CENPC;H3C6;H4C3;H2BC5;CENPQ;TUBB2B;TUBB2A;H4C12;H2BC14;MAD1L1;RAC1;H2AZ2;NCF1;CTTN;RHOG;NUP98;PPP2R5E;MAPK3;SPC24;DSN1*	6q21, 7p22.1, 16p11.2, 4q13.2, 6p22.2, 6p12.3, 7p13, 11q13.3, 11p15.4, 19p13.2, 1q32.3, 1q44, 6p25.2, 6p22.1, 7p22.3, 11p15.4, 14q23.2, 20q11.23, 7q11.23	3.08E-05
	Reactome signaling by Rho GTPases	*WASF2;PPP2R5A;AHCTF1;CENPC;ARAP2;H3C6;H4C3;H2BC5;CENPQ;TUBB2B;TUBB2A;H4C12;H2BC14;MAD1L1;RAC1;H2AZ2;NCF1;ARHGEF35;ARHGEF5;DLC1;CTTN;RHOG;NUP98;PPP2R5E;MAPK3;SPC24;DSN1*	6q21, 7p22.1, 16p11.2, 4q13.2, 6p22.2, 6p12.3, 7p13, 7q35, 8p22, 11q13.3, 11p15.4, 19p13.2, 1q32.3, 1q44, 6p25.2, 6p22.1, 7p22.3, 11p15.4, 14q23.2, 20q11.23, 7q11.23, 4p14, 7q35	8.11E-05
	BioCarta MAPK pathway	*MAP3K6;SHC1;MAP3K7;RIPK1;MAPK13;RAC1;MAP3K11;RPS6KA5;MAPK3*	1p36.11, 7p22.1, 1q21.3, 16p11.2, 6q15, 6p25.2, 6p21.31, 11q13.1, 14q32.11	0.00499

a Fisher’s exact test *p*-value represents the adjusted *p*-value for genes in the pathway using Fisher’s exact test that are adjusted by Benjamini & Hochberg correction method.

Furthermore, the AT1R pathway is responsible for promoting hypertension, G protein-dependent signaling, transactivation of growth factor receptors, NADPH oxidase, and ROS signaling explaining why the *RAC1* gene was enriched by the AT1R pathway (Kawai et al., 2017). In addition to the *RAC1* gene, the *PPP2R5A* gene in the tissue whole blood paired with the Reactome cell cycle pathway was hit on. The *PPP2R5A* gene stands for protein phosphatase 2 regulatory subunit B’alpha. The gene serves as a subunit of the protein phosphatase 2A (PP2A) holoenzyme, which plays an essential role in regulating a diverse array of myocyte functions through dephosphorylation of target molecules. Functioning as an important phosphatase, the PP2A holoenzyme is critical for serving as a regulatory module within the heart, such that dysregulation of PP2A function may contribute to cardiac diseases. Alterations in PP2A activity are associated with heart failure and arrhythmia (Lubbers and Mohler, 2016). The varying types of myocardial infarction make it difficult for researchers to pinpoint a cure. In recent years, scientists have recognized multiple types of myocardial infarction with different causes, yet the knowledge of its pathogenic mechanisms is still poorly understood and greatly lacking (DeFilippis et al., 2019). While the different causes of myocardial infarction can be difficult to pinpoint, we can start by identifying the pathways, tissues, genes that are related to the causes. The results have shown some genomic mechanisms contributing to myocardial infarction, whether it be the enrichment of the RAC1 protein leading to the regulation of NADPH oxidase causing heart failure, or the altered regulation in the PP2A gene leading to heart failure and arrhythmia. The importance of these findings is two-fold: first, these results could serve as a pipeline to benefit the scientific community through reducing repeated work, and second, the discovered specific pathway-tissue-gene results could help researchers to reveal pathogenesis mechanisms in myocardial infarction in hopes to lower its occurrence rates or raise the rates of survival.

## 4 Discussion

We have extended the loci2path (Xu et al., 2020) by using the latest multi-tissue eQTLs data set from GTEx V8 release and adding PID, Reactome, and WikiPathways databases. The total numbers of eQTLs for each of 49 tissues we used in this study are shown in Supplementary Table S2. Our results of enrichment analysis have suggested multiple novel biological hypotheses of disease mechanisms for AD, PD, and schizophrenia. The proposed mechanisms of the increase of caspase-3 level in amygdala tissue and KMO production that may contribute to AD’s memory loss symptoms by increasing apoptosis and neuronal loss and decreasing kynurenine metabolite levels were supported by multiple past studies. The impaired lysosomal functions of GCase, lysosomal-associated membrane protein 2A, and heat shock cognate 70 resulted from mutations in genes corresponding to these proteins may cause α-synuclein accumulation to begin and thus lead to PD. The interaction among *HLA-C*, *HLA-DRB1*, and *HLA-DQB1* is likely to take part in schizophrenia’s pathogenesis as well.

Our study has extensively evaluated multiple gene pathways’ involvements in the ten traits and further investigated significant genes in each pathway that were hit in the given genomic query regions. The proposed hypotheses have opened new avenues to explore the underlying molecular mechanisms and thus could illuminate further investigations on these traits. We have also found many interesting associations between eQTLs and gene pathways at trait-associated variants of NSCLC, blood pressure, autism spectrum disorder, and myocardial infarction which provided valuable insights into our comprehensive understandings of them. Furthermore, our study has confirmed the advantages of using tissue-specific eQTLs enrichment analysis at pathway level, because our findings based on loci2path software were strongly supported by multiple previous studies (Xu et al., 2020). This has indicated that using eQTLs catalogs to find links between genomic loci and their corresponding eGenes is valid and should be vastly applied in future studies involving gene sets and traits.

There were several limitations in our study. Due to the nature of the statistical analysis, our findings from loci2path could not be considered as providing direct understandings of biological mechanisms underpinning these traits, and we were only able to generate hypotheses for trait determination. These hypotheses should be experimentally verified by conducting further in-depth functional studies by molecular biology laboratories. In addition, loci2path′s reliance on current eQTLs sets data from GTEx could also lead to biased results since the eQTLs sets data from brain tissues were significantly smaller than other tissues like tibial nerves, leg skin without sun exposure, and thyroid. This was caused by the limited sample sizes of brain tissues from GTEx, which may result in missing important biological pathways in brain tissues for neurodegenerative diseases due to inadequate statistical power. The imbalance of eQTLs sizes of various tissues could also bring false-positive results in tissues with more samples and generate coincidental enrichment of certain pathways at tissues not related to the traits. Therefore, results from loci2path need to be treated with extra care, and only the most significant tissue-pathway associations should be extracted for analysis with sufficient past evidence. The software itself also has rooms for improvement, like including new gene pathway sets and adding annotations on pathways uniquely enriched in a tissue.

Future studies on neurodegenerative diseases specifically should implement more data on brain tissues to increase the accuracy of loci2path. Other neurodegenerative diseases like bipolar disorder and attention deficit disorder could be added for a systematic analysis on their patterns to find potential patterns for commonality among this type of disease.

## Data Availability

The original contributions presented in the study are included in the article/Supplementary Material, further inquiries can be directed to the corresponding author.

## Abbreviations

AD, Alzheimer’s disease; ARHGDIB, Rho GDP dissociation inhibitor beta; Aß, amyloid-beta; DCM, dilated cardiomyopathy; eGene, target gene of an expression quantitative trait locus; eQTL, expression quantitative trait locus; GCase, glucocerebrosidase; HLA-C, major histocompatibility complex, Class I, C; HLA-DQB1, major histocompatibility complex, Class II, DQ Beta 1; HLA-DRB1, major histocompatibility complex, Class II, DR Beta 1; ICM, ischemic cardiomyopathy; IL-17, interleukin-17; IL-37, interleukin-37; ILC, innate lymphoid cell; KMO, kynurenine 3-monooxygenase; NSCLC, non-small cell lung cancer; PD, Parkinson’s disease; PP2A, protein phosphatase 2A; PPP2R5A, protein phosphatase 2 regulatory subunit B’alpha; ROS, reactive oxygen species.
