# Supplementary material for: Systematic Exploration in Tissue-Pathway Associations of Complex Traits Using Comprehensive eQTLs Catalog
Source: Front Big Data. 2021 Nov 3;4:719737. doi: 10.3389/fdata.2021.719737 (PMC8595594; doi:10.3389/fdata.2021.719737)

Supplementary Materials

for

Systematic Exploration in Tissue-Pathway Associations of Complex Traits Using Comprehensive eQTLs Catalog

Boqi Wang^1^, James Yang^2^, Steven Qiu^3^, Yongsheng Bai^4^, Zhaohui Qin^5*^.

^1^Emory University, Atlanta, Georgia, 30322, USA

^2^Carmel High School, Carmel, Indiana, 46032, USA

^3^James Martin High School, Arlington, Texas, 76016, USA

^4^Next-Gen Intelligent Science Training, Ann Arbor, Michigan, 48105, USA

^5^Department of Biostatistics and Bioinformatics, Emory University, Atlanta, Georgia, 30322, USA

Supplementary Figure 1. Heatmaps of Parkinson’s disease’s eQTLs enrichment results in (A) KEGG and (B) WikiPathways pathway sets, respectively.


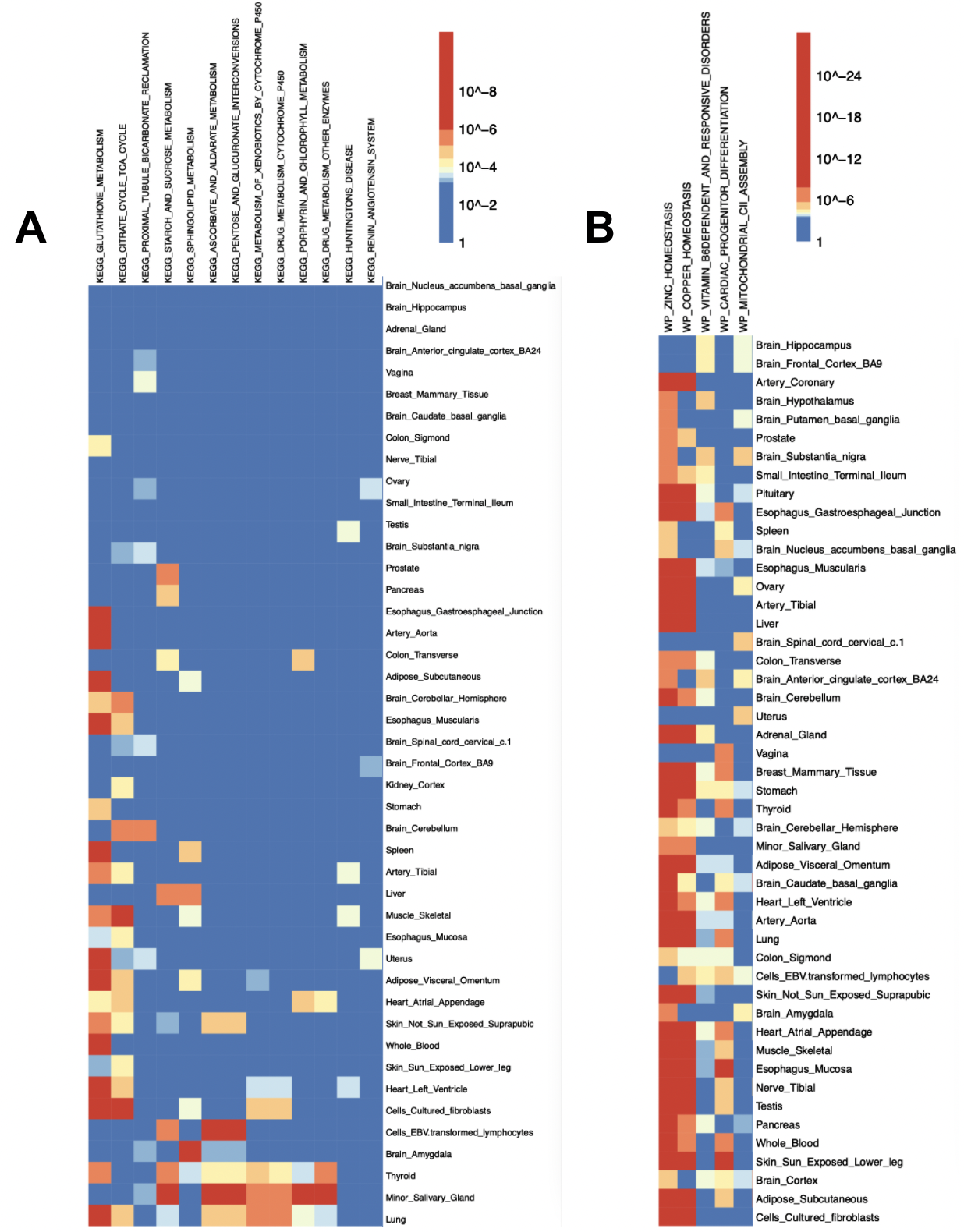


Supplementary Figure 2. Heatmap of non-small cell lung cancer’s eQTLs enrichment results in the BioCarta pathway set.


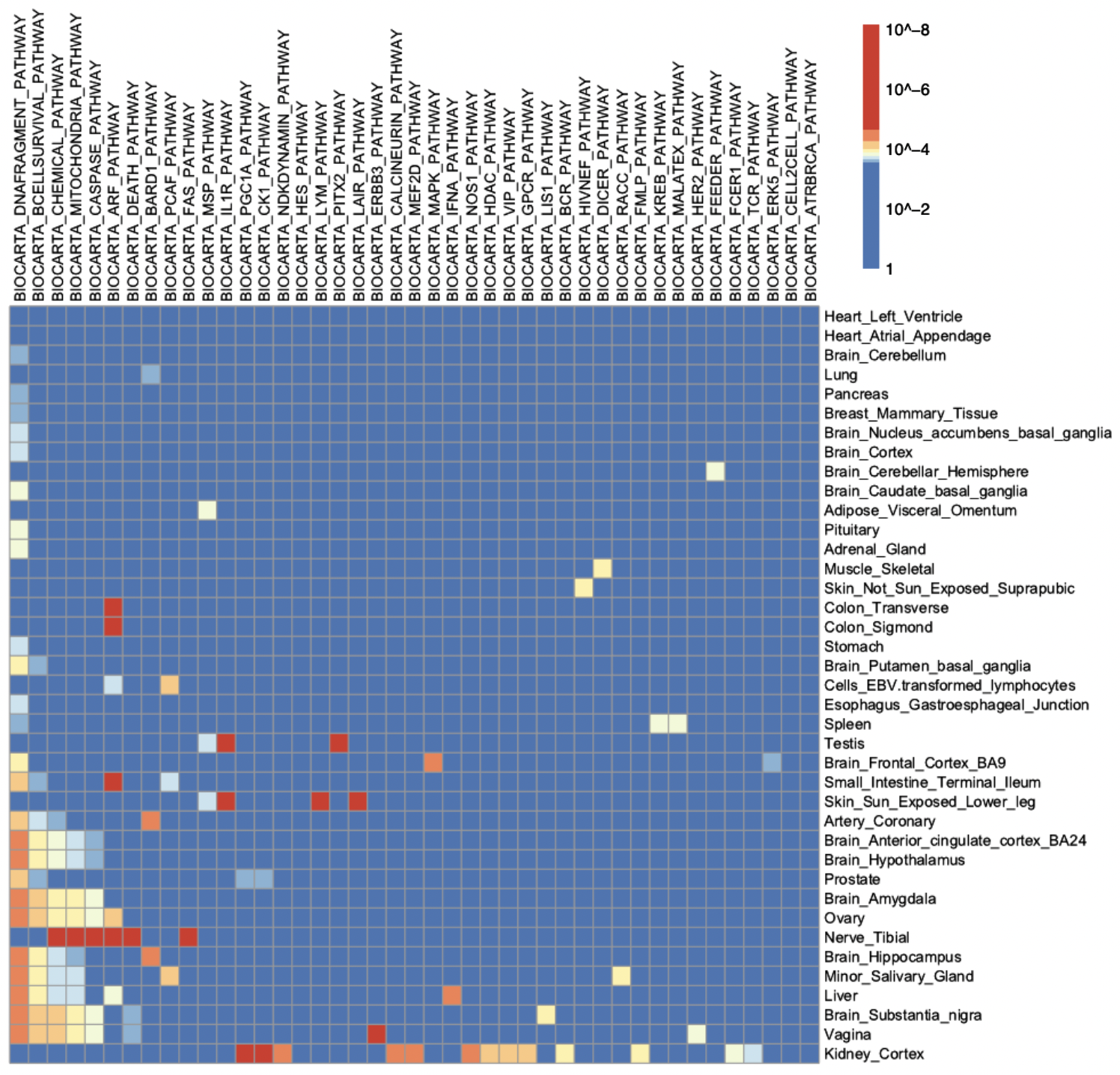


Supplementary Figure 3. Heatmap of autism spectrum disorder’s eQTLs enrichment results in the BioCarta pathway set.


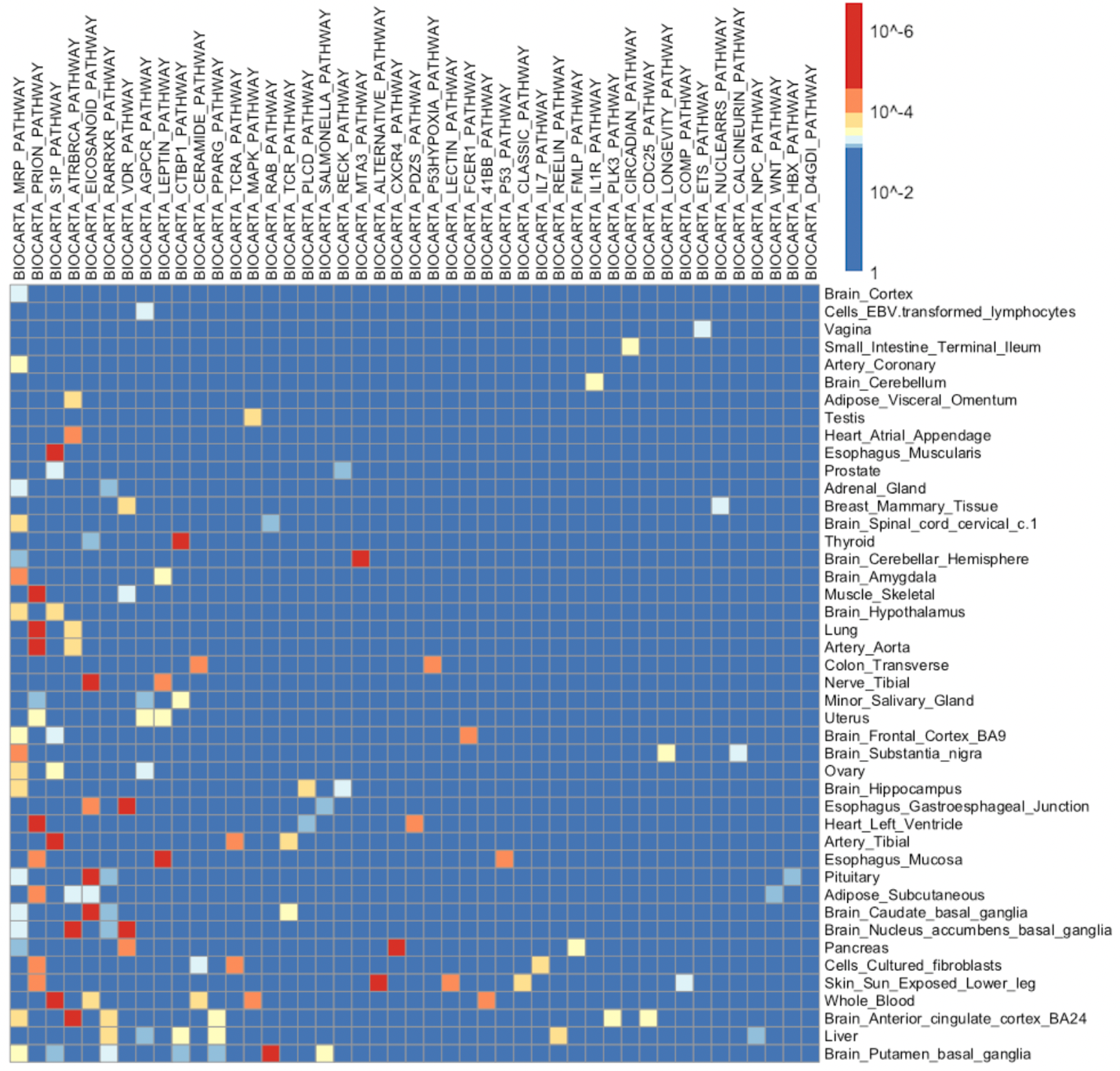


Supplementary Figure 4. Heatmap of autism spectrum disorder’s eQTLs enrichment results in the KEGG pathway set.


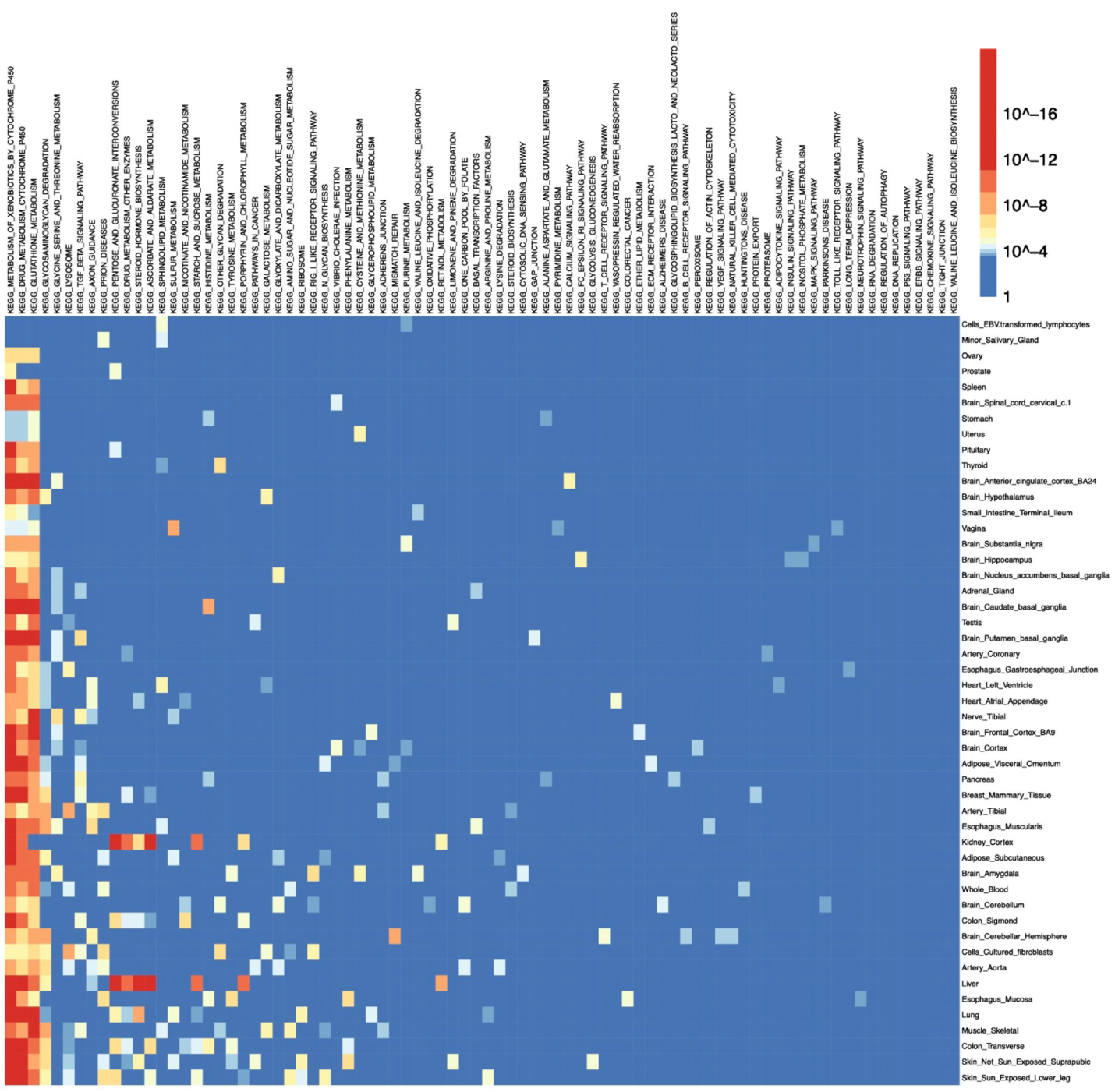


Supplementary Figure 5. Heatmap of autism spectrum disorder’s eQTLs enrichment results in the PID pathway set.


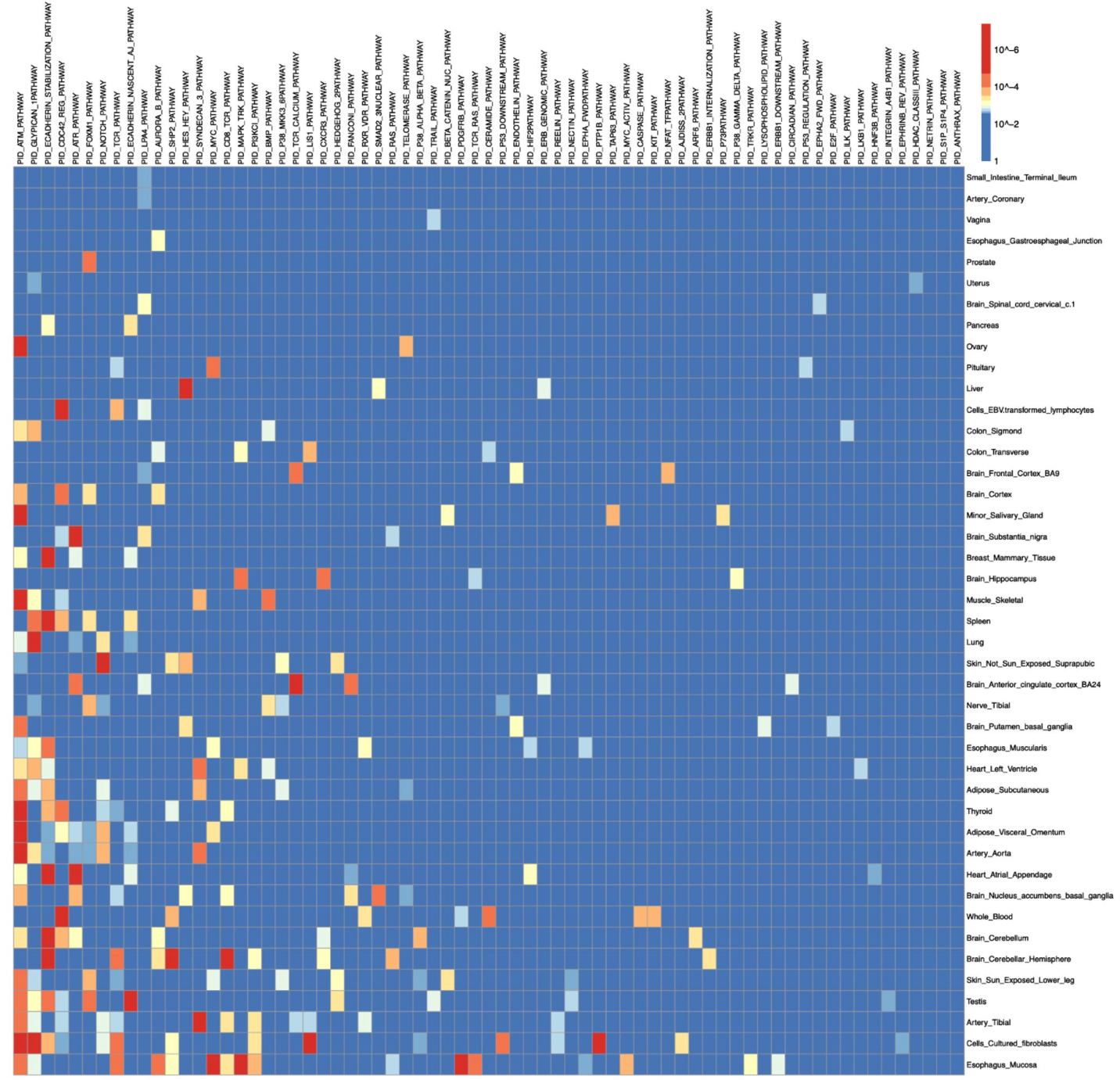


Supplementary Figure 6. Heatmap of autism spectrum disorder’s eQTLs enrichment results in the Reactome pathway set.

Supplementary Figure 7. Heatmap of autism spectrum disorder’s eQTLs enrichment results in the WikiPathways pathway set.


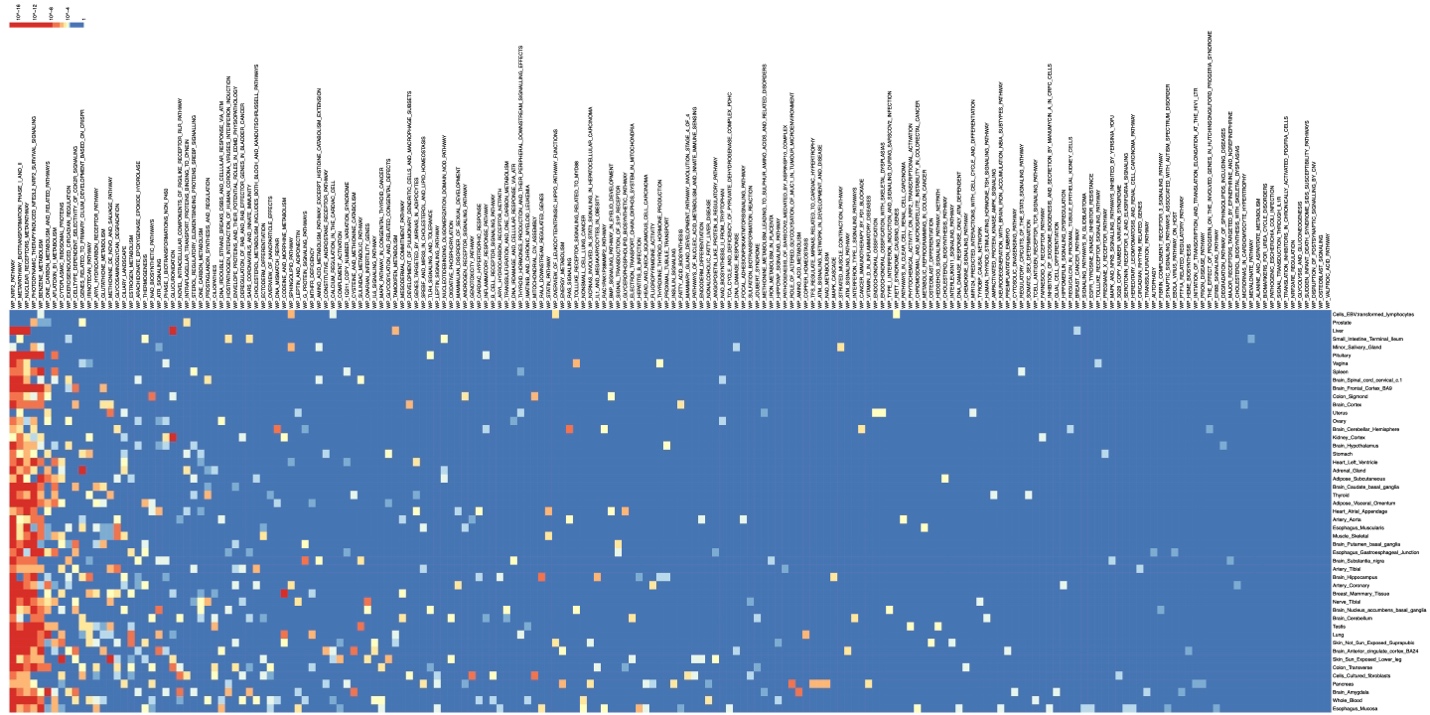


Supplementary Figure 8. Heatmaps of myocardial infarction’s eQTLs enrichment results in (A) BioCarta and (B) Reactome pathway sets, respectively.


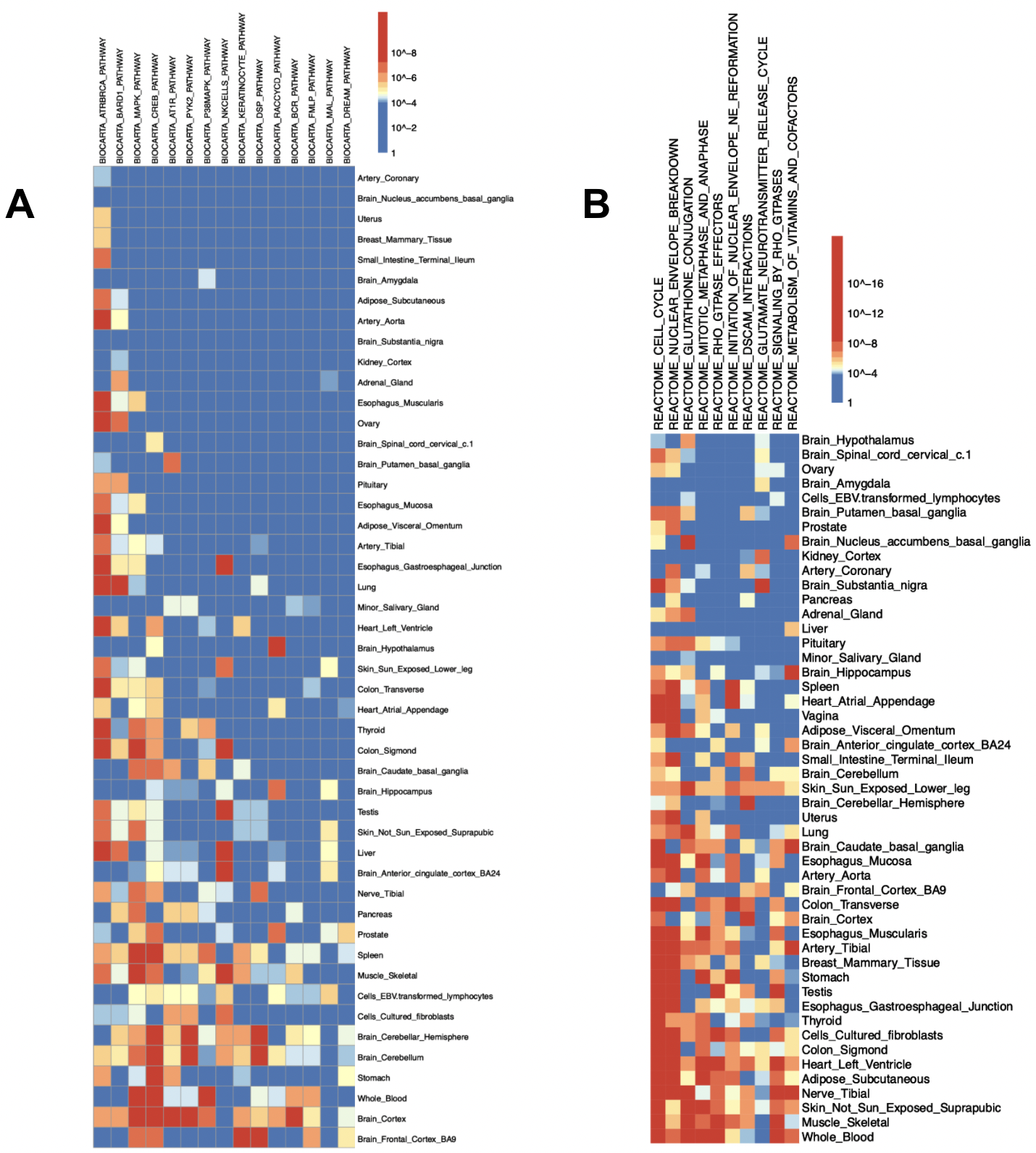

Supplement: Supplementary file 4 [file DataSheet2.DOCX]
